# Supplementary material for: RNA selectively modulates activity of virulent amyloid PSMα3 and host-defense LL-37 via phase separation and aggregation dynamics
Source: eLife. 2026 Jul 20;15:RP109290. doi: 10.7554/eLife.109290 (PMC13384500; doi:10.7554/eLife.109290)
Supplement: Figure 1—source data 1. [file elife-109290-fig1-data1.zip › Figure 1-source data 1/Figure 1-source data 1.pdf]

Figure 1–source data 1

Single stranded RNA – Poly(A) RNA

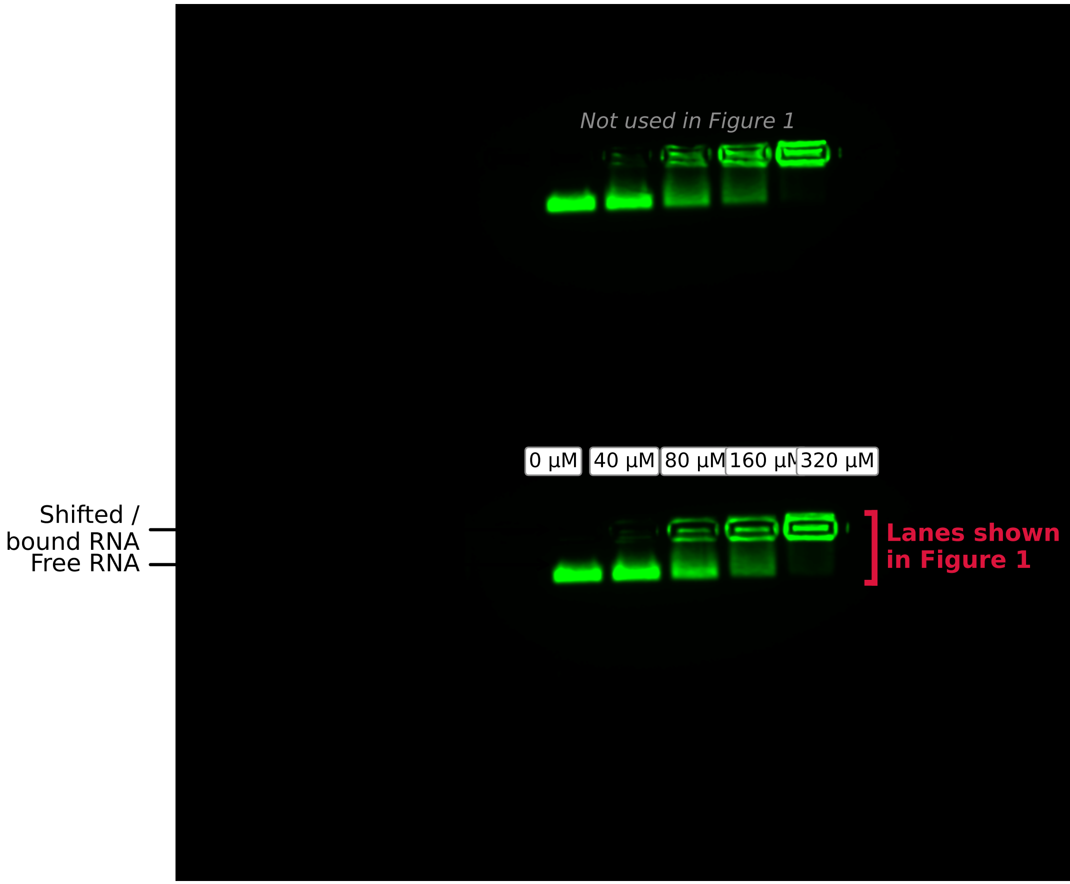

Full uncropped IR800CW EMSA gel. Source file: PSMa3_ssRNA_300126_2.tif. Only the gel row used in Figure 1 is labelled; the other row in the same scan is a separate experiment.

Figure 1–source data 1

Double stranded RNA – Poly(AU) RNA

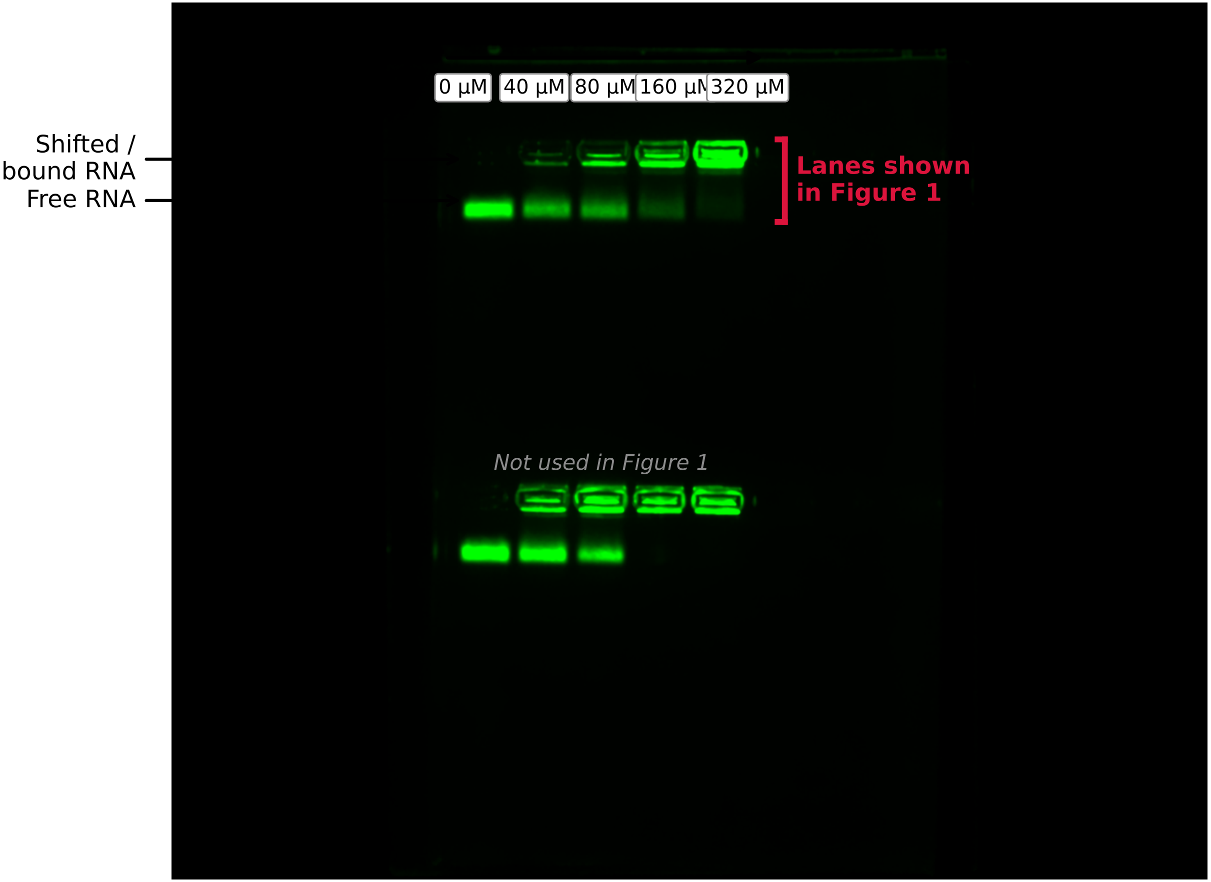

Full uncropped IR800CW EMSA gel. Source file: PSMa3_EGCG_CTRL_dsRNA_ir800.tif (28.7.24). Only the gel row used in Figure 1 is labelled; the other row in the same scan is a separate experiment.
