# Supplementary figures and images for: RNA selectively modulates activity of virulent amyloid PSMα3 and host-defense LL-37 via phase separation and aggregation dynamics
Source: eLife. 2026 Jul 20;15:RP109290. doi: 10.7554/eLife.109290 (PMC13384500; doi:10.7554/eLife.109290)

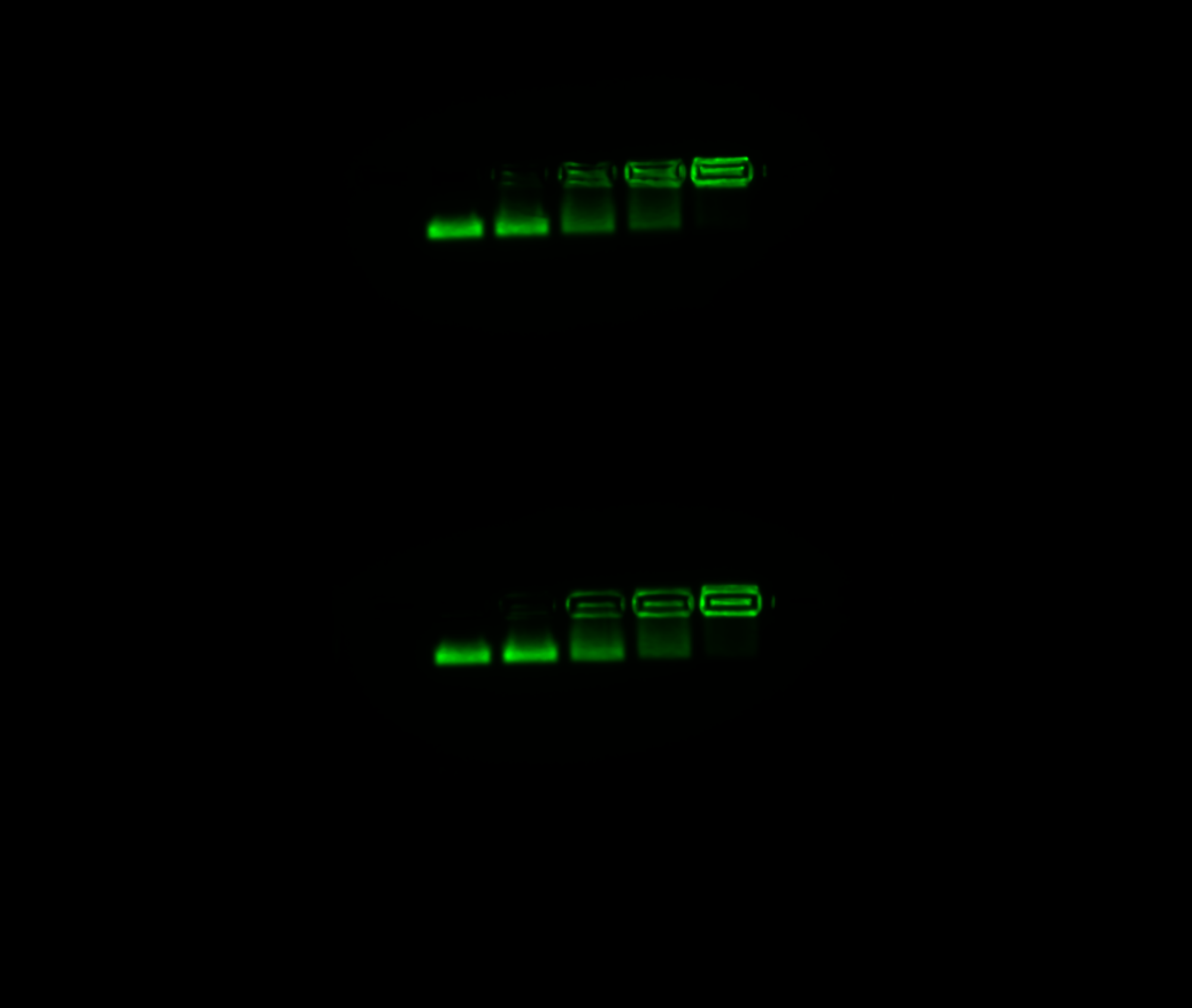

Supplement: Figure 1—source data 2. [file elife-109290-fig1-data2.zip › Figure 1-source data 2/Figure 1 _ PSMa3 with PolyA.tif]

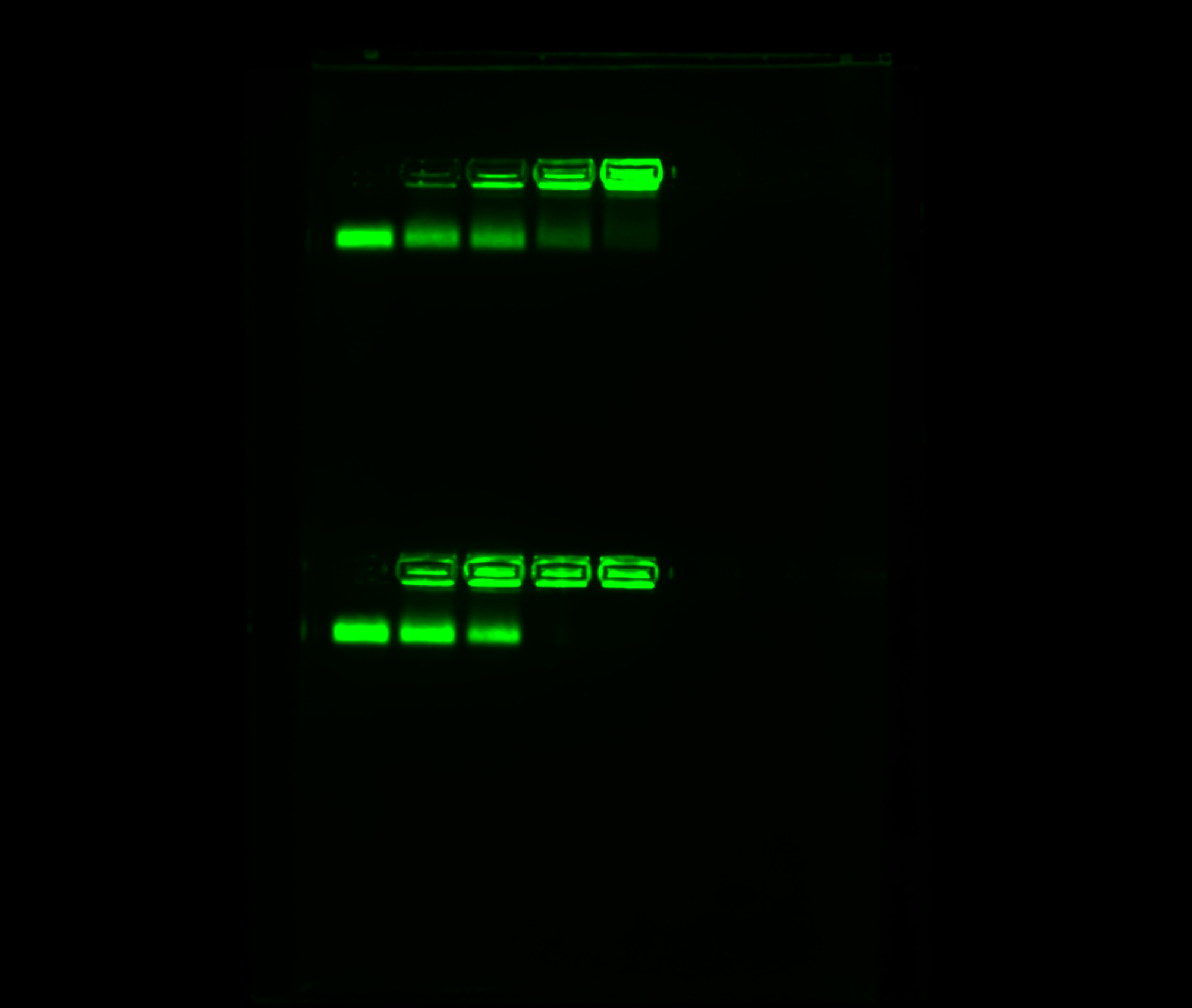

Supplement: Figure 1—source data 2. [file elife-109290-fig1-data2.zip › Figure 1-source data 2/Figure 1 _ PSMa3 with PolyAU.tif]
