# Supplementary material for: RNA selectively modulates activity of virulent amyloid PSMα3 and host-defense LL-37 via phase separation and aggregation dynamics
Source: eLife. 2026 Jul 20;15:RP109290. doi: 10.7554/eLife.109290 (PMC13384500; doi:10.7554/eLife.109290)
Supplement: MDAR checklist [file elife-109290-mdarchecklist1.docx]

**Materials Design Analysis Reporting (MDAR)**

**Checklist for Authors — eLife**

**Manuscript:** *RNA Selectively Modulates Activity of Virulent Amyloid PSMα3 and Host Defense LL-37 via Phase Separation and Aggregation Dynamics*

Rayan et al. | eLife submission

| **Item** | **Indicate where provided: section/figure legend** | **N/A** |
| --- | --- | --- |
| **MATERIALS** | | |
| ***Newly created materials*** | | |
| The manuscript includes a dedicated "materials availability statement" providing transparent disclosure about availability of newly created materials including details on how materials can be accessed and describing any restrictions on access. | Materials and Methods, Key Resources Table, and Peptide and Poly(AU) RNA preparation section. Peptides were custom-synthesized (GL Biochem) and RNA obtained commercially (Sigma-Aldrich; IDT); no restrictions on access. No newly created (e.g., mutant) materials were generated. |  |
|  | | |
| ***Antibodies*** | | |
| For commercial reagents, provide supplier name, catalogue number and RRID, if available. | Key Resources Table: all reagents listed with supplier, catalog number, and RRID where available. |  |
|  | | |
| ***DNA and RNA sequences*** | | |
| Short novel DNA or RNA including primers, probes: Sequences should be included or deposited in a public repository. |  | **✓** |
|  | | |
| ***Cell materials*** | | |
| Cell lines: Provide species information, strain. Provide accession number in repository OR supplier name, catalog number, clone number, OR RRID. | Key Resources Table and Materials and Methods (LDH cytotoxicity section): HeLa cells, Homo sapiens, ATCC® CCL-2™, RRID:CVCL_0030. Human cervical carcinoma cell line; kind gift from Prof. Michael H. Glickman, Technion. |  |
| Primary cultures: Provide species, strain, sex of origin, genetic modification status. |  | **✓** |
|  | | |
| ***Experimental animals*** | | |
| Laboratory animals or Model organisms: Provide species, strain, sex, age, genetic modification status. Provide accession number in repository OR supplier name, catalog number, clone number, OR RRID. |  | **✓** |
| Animal observed in or captured from the field: Provide species, sex, and age where possible. |  | **✓** |
|  | | |
| ***Plants and microbes*** | | |
| Plants: provide species and strain, ecotype and cultivar where relevant, unique accession number if available, and source (including location for collected wild specimens). |  | **✓** |
| Microbes: provide species and strain, unique accession number if available, and source. | Key Resources Table and Materials and Methods (Bacterial Viability section): Escherichia coli strain RFM795 (lptD4213), CGSC #14179, Yale University, obtained as a kind gift from Prof. Sima Yaron (Technion). Carries an in-frame deletion in lptD (D330–D352); increased outer membrane permeability. |  |
|  | | |
| ***Human research participants*** | | |
| If collected and within the bounds of privacy constraints report on age, sex, gender and ethnicity for all study participants. |  | **✓** |
| **DESIGN** | | |
| ***Study protocol*** | | |
| If the study protocol has been pre-registered, provide DOI. For clinical trials, provide the trial registration number OR cite DOI. |  | **✓** |
|  | | |
| ***Laboratory protocol*** | | |
| Provide DOI OR other citation details if detailed step-by-step protocols are available. | Detailed step-by-step protocols provided in the Materials and Methods section; no separate protocol DOI. |  |
|  | | |
| ***Experimental study design (statistics details) — For in vivo studies: State whether and how the following have been done*** | | |
| Sample size determination |  | **✓** |
| Randomisation |  | **✓** |
| Blinding |  | **✓** |
| Inclusion/exclusion criteria |  | **✓** |
|  | | |
| ***Sample definition and in-laboratory replication*** | | |
| State number of times the experiment was replicated in the laboratory. | Materials and Methods (each assay subsection) and figure legends: all experiments performed in at least three independent biological replicates on separate days. |  |
| Define whether data describe technical or biological replicates. | Materials and Methods and figure legends: data are from at least three independent biological replicates, each assay performed in technical triplicate per experiment. |  |
|  | | |
| ***Ethics*** | | |
| Studies involving human participants: State details of authority granting ethics approval (IRB or equivalent committee(s)), provide reference number for approval. |  | **✓** |
| Studies involving experimental animals: State details of authority granting ethics approval (IRB or equivalent committee(s)), provide reference number for approval. |  | **✓** |
| Studies involving specimen and field samples: State if relevant permits obtained, provide details of authority approving study; if none were required, explain why. |  | **✓** |
|  | | |
| ***Dual Use Research of Concern (DURC)*** | | |
| If study is subject to dual use research of concern regulations, state the authority granting approval and reference number for the regulatory approval. |  | **✓** |
| **ANALYSIS** | | |
| ***Attrition*** | | |
| Describe whether exclusion criteria were pre-established. Report if sample or data points were omitted from analysis. If yes, report if this was due to attrition or intentional exclusion and provide justification. |  | **✓** |
|  | | |
| ***Statistics*** | | |
| Describe statistical tests used and justify choice of tests. | Materials and Methods (each assay subsection) and figure legends (Figs. 4, 6, 8, and figure supplements): one-way ANOVA for normally distributed data, GraphPad Prism v11. Kruskal-Wallis test with Dunn's post hoc test and Bonferroni correction for non-parametric data (Fig. 3-figure supplement 1). Significance thresholds: *p<0.05, **p<0.01, ***p<0.001, ****p<0.0001. |  |
|  | | |
| ***Data availability*** | | |
| For newly created and reused datasets, the manuscript includes a data availability statement that provides details for access (or notes restrictions on access). | Data availability statement (end of Materials and Methods): raw data deposited at Zenodo, https://doi.org/10.5281/zenodo.17598867. |  |
| When newly created datasets are publicly available, provide accession number in repository OR DOI and licensing details where available. | Zenodo repository: https://doi.org/10.5281/zenodo.17598867. |  |
| If reused data is publicly available provide accession number in repository OR DOI, OR URL, OR citation. |  | **✓** |
|  | | |
| ***Code availability*** | | |
| For any computer code/software/mathematical algorithms essential for replicating the main findings of the study, the manuscript includes a data availability statement that provides details for access or notes restrictions. | Data availability statement: in-house Scilab 2025.0.0 scripts used for NMR signal integration deposited at Zenodo, https://doi.org/10.5281/zenodo.17598867. |  |
| Where newly generated code is publicly available, provide accession number in repository, OR DOI OR URL and licensing details where available. | Custom Scilab scripts deposited at Zenodo: https://doi.org/10.5281/zenodo.17598867. |  |
| If reused code is publicly available provide accession number in repository OR DOI OR URL, OR citation. |  | **✓** |
| **REPORTING** | | |
| ***Adherence to community standards*** | | |
| State if relevant guidelines (e.g., ICMJE, MIBBI, ARRIVE, STRANGE) have been followed, and whether a checklist (e.g., CONSORT, PRISMA, ARRIVE) is provided with the manuscript. |  | **✓** |

**Note:** Items marked N/A (✓) are not applicable to this study, which uses no animal models, clinical participants, pre-registered protocols, or field specimens. All statistical methods are described in the Materials and Methods section and figure legends.
