# Supplementary material for: RNA selectively modulates activity of virulent amyloid PSMα3 and host-defense LL-37 via phase separation and aggregation dynamics
Source: eLife. 2026 Jul 20;15:RP109290. doi: 10.7554/eLife.109290 (PMC13384500; doi:10.7554/eLife.109290)
Supplement: Peer review history

# Editor report: eLife Assessment

**DOI:** 10.7554/eLife.109290.4.sa0

**Authors:** Feng Ding

**Affiliations:** Clemson University United States

**Keywords:** Solid, Valuable

This study presents **valuable** findings on the differential effects of RNA on the phase separation, aggregation dynamics, and bioactivity of PSMα3 and LL-37. The authors provide **solid** evidence from complementary biophysical and cell-based experiments that RNA influences peptide assembly and associated in vitro activities. The study is of interest for understanding interactions between amyloidogenic peptides and nucleic acids.

# Referee report: Reviewer #1 (Public review)

**DOI:** 10.7554/eLife.109290.4.sa1

[Editors' note: This version has been assessed by the Reviewing Editor without further input from the original reviewers. The authors have addressed the comments raised in the previous round of review.]

Summary:

The manuscript by Rayan et al. aims to elucidate the role of RNA as a context-dependent modulator of liquid-liquid phase separation (LLPS), aggregation, and bioactivity of the amyloidogenic peptides PSMα3 and LL-37, motivated by their structural and functional similarities.

Strengths:

The authors combine extensive biophysical characterization with cell-based assays to investigate how RNA differentially regulates peptide aggregation states and associated cytotoxic and antimicrobial functions.

# Referee report: Reviewer #2 (Public review)

**DOI:** 10.7554/eLife.109290.4.sa2

In this paper, Rayan et al. report that RNA influences cytotoxic activity of the staphylococcal secreted peptide cytolysin PSMalpha3 versus human cells and *E. coli* by impacting its aggregation. The authors used sophisticated methods of structural analysis and describe the associated liquid-liquid phase separation. They also compare to the influence of RNA on aggregation and activity of LL-37, which shows differences to that on PSMalpha3.

Major comments on the previous version:

(1) The premise, as stated in the introduction and elsewhere, that PSMalpha3 amyloids are biologically functional, is highly debatable and has never been conclusively substantiated. The property that matters most for the present study, cytotoxicity, is generally attributed to PSM monomers, not amyloids. The likely erroneous notion that PSM amyloids are the predominant cytotoxic form is derived from an earlier study by the authors that has described a specific amyloid structure of aggregated PSMalpha3. Other authors have later produced evidence that, quite unsurprisingly, indicated that aggregation into amyloids decreases, rather than increases, PSM cytotoxicity. Unfortunately, yet other groups have in the meantime published in-vitro studies on "functional amyloids" by PSMs without critically challenging the concept of PSM amyloid "functionality". Of note, the authors' own data in the present study that show strongly decreased cytotoxicity of PSMalpha3 after prolonged incubation are in agreement with monomer-associated cytotoxicity as they can be easily explained by the removal of biologically active monomers from the solution.

In their revision and in the rebuttal, the authors have further described their concept regarding what they call "functionality" of PSMalpha3 amyloids. They now admit that monomers are the active cytolytic form, like other researchers have stressed, whereas amyloids are not. This represents a considerable difference to earlier papers in which they ascribed functionality, i.e. cytolytic capacity, to PSMalpha3 amyloids, a claim that has raised considerable controversy. Now, they use the term "functional " to describe that PSMalpha3 amyloids, while not cytolytic, can be reversed to a cytolytic monomeric state, calling them a "dynamic reservoir". There is no evidence that such a reservoir is necessary for the cytolytic activity of the monomers to be established; also, there is no evidence that in a biological system, such an amyloid reservoir exists. To continue calling PSMalpha3 amyloids "functional" based on this - considerably changed - concept of the authors appears inappropriate, given the finally admitted absence of cytolytic activity of the PSM amyloids in addition to the continuing complete lack of evidence of any biological relevance of PSM amyloid formation.

(2) That RNA may interfere with PSM aggregation and influence activity is not very surprising, given that PSM attachment to nucleic acids - while not studied in as much detail as here - has been described. Importantly, it does not become clear whether this effect has biologically significant consequences beyond influencing, again not surprisingly, cytotoxicity in vitro. The authors do show in nice microscopic analyses that labeled PSMalpha3 attaches to nuclei when incubated with HeLa cells. However, given that the cells are killed rapidly by membrane perturbation by the applied PSM concentrations, it remains unclear and untested whether the attachment to nucleic acids in dying cells makes any contribution to PSM-induced cell death or has any other biological significance.

# Author comment: Author response

**DOI:** 10.7554/eLife.109290.4.sa3

**Authors:** Bader Rayan, Eilon Barnea, Rinat Indig, Christian Felipe Pantoja, Jesse Gayk, Yael Lupu-Haber, Alexander Upcher, Amir Argoetti, Jacob Aunstrup Larsen, Alexander Kai Buell, Markus Zweckstetter, Meytal Landau

**Affiliations:** Technion - Israel Institute of Technology Haifa Israel; Technion - Israel Institute of Technology Haifa Israel; Technion - Israel Institute of Technology Haifa Israel; German Center for Neurodegenerative Diseases Göttingen Germany; Centre for Structural Systems Biology Hamburg Germany; Technion - Israel Institute of Technology Haifa Israel; Ben-Gurion University of the Negev Beer-Sheva Israel; Technion - Israel Institute of Technology Haifa Israel; Technical University of Denmark Kongens Lyngby Denmark; Technical University of Denmark Kgs Lyngby Denmark; German Center for Neurodegenerative Diseases Göttingen Germany; Deutsches Elektronen-Synchrotron DESY Hamburg Germany

The following is the authors’ response to the previous reviews

**eLife Assessment**

This study presents valuable findings on the differential effects of RNA on the phase separation, aggregation dynamics, and bioactivity of PSMα3 and LL-37. The authors provide solid evidence from complementary biophysical and cell-based experiments that RNA influences peptide assembly and associated in vitro activities. The study is of interest for understanding interactions between amyloidogenic peptides and nucleic acids, although the physiological significance and some aspects of the mechanistic interpretation would benefit from further clarification.

We are grateful for the positive assessment. The two outstanding concerns about physiological significance and mechanistic interpretation are addressed in detail below through Reviewer #2's comments. We have made targeted revisions throughout the manuscript, and have been careful to distinguish genuine clarifications from reframing that would misrepresent what the data show.

**Public Reviews:**

**Reviewer #1 (Public review):**

Summary:

The manuscript by Rayan et al. aims to elucidate the role of RNA as a contextdependent modulator of liquid-liquid phase separation (LLPS), aggregation, and bioactivity of the amyloidogenic peptides PSMα3 and LL-37, motivated by their structural and functional similarities.

Strengths:

The authors combine extensive biophysical characterization with cell-based assays to investigate how RNA differentially regulates peptide aggregation states and associated cytotoxic and antimicrobial functions.

Weaknesses:

While the study addresses an interesting and timely question with potentially broad implications for host-pathogen interactions and amyloid biology, some aspects of the experimental design and data analysis require further clarification and strengthening.

We thank Reviewer #1 for the positive assessment. Previous revision round incorporated all major quantitative additions requested:

Quantitative EMSA binding analysis with Kd values and Hill coefficients (Fig. S1)

Quantitative FRAP recovery curves with mobile fractions and half-times (Figs. S4, S8, S12)

Colocalization metrics — Pearson's correlation coefficient and Manders' overlap coefficients (Fig. S5)

Quantification of AmyTracker630 amyloid signal intensity (Fig. S6)

Explicit acknowledgment of limitations regarding phase diagram boundaries and csat

Revised interpretation clarifying nucleolar localization as phenomenological, not causal

**Reviewer #2 (Public review):**

In this paper, Rayan et al. report that RNA influences cytotoxic activity of the staphylococcal secreted peptide cytolysin PSMalpha3 versus human cells and *E. coli* by impacting its aggregation. The authors used sophisticated methods of structural analysis and describe the associated liquidliquid phase separation. They also compare to the influence of RNA on aggregation and activity of LL-37, which shows differences to that on PSMalpha3.

That RNA impacts PSM cytotoxicity when co-incubated in vitro becomes clear. However, I have two major problems with this study:

The premise, as stated in the introduction and elsewhere, that PSMalpha3 amyloids are biologically functional, is highly debatable and has never been conclusively substantiated. The property that matters most for the present study, cytotoxicity, is generally attributed to PSM monomers, not amyloids. The likely erroneous notion that PSM amyloids are the predominant cytotoxic form is derived from an earlier study by the authors that has described a specific amyloid structure of aggregated PSMalpha3. Other authors have later produced evidence that, quite unsurprisingly, indicated that aggregation into amyloids decreases, rather than increases, PSM cytotoxicity. Unfortunately, yet other groups have in the meantime published in-vitro studies on "functional amyloids" by PSMs without critically challenging the concept of PSM amyloid "functionality". Of note, the authors' own data in the present study that show strongly decreased cytotoxicity of PSMalpha3 after prolonged incubation are in agreement with monomerassociated cytotoxicity as they can be easily explained by the removal of biologically active monomers from the solution.

In their revision and in the rebuttal, the authors have further described their concept regarding what they call "functionality" of PSMalpha3 amyloids. They now admit that monomers are the active cytolytic form, like other researchers have stressed, whereas amyloids are not. This represents a considerable difference to earlier papers in which they ascribed functionality, i.e. cytolytic capacity, to PSMalpha3 amyloids, a claim that has raised considerable controversy. Now, they use the term "functional " to describe that PSMalpha3 amyloids, while not cytolytic, can be reversed to a cytolytic monomeric state, calling them a "dynamic reservoir". There is no evidence that such a reservoir is necessary for the cytolytic activity of the monomers to be established; also, there is no evidence that in a biological system, such an amyloid reservoir exists. To continue calling PSMalpha3 amyloids "functional" based on this - considerably changed - concept of the authors appears inappropriate, given the finally admitted absence of cytolytic activity of the PSM amyloids in addition to the continuing complete lack of evidence of any biological relevance of PSM amyloid formation.

That RNA may interfere with PSM aggregation and influence activity is not very surprising, given that PSM attachment to nucleic acids - while not studied in as much detail as here - has been described. Importantly, it does not become clear whether this effect has biologically significant consequences beyond influencing, again not surprisingly, cytotoxicity in vitro. The authors do show in nice microscopic analyses that labeled PSMalpha3 attaches to nuclei when incubated with HeLa cells. However, given that the cells are killed rapidly by membrane perturbation by the applied PSM concentrations, it remains unclear and untested whether the attachment to nucleic acids in dying cells makes any contribution to PSM-induced cell death or has any other biological significance. Overall, the findings can be explained in a much more straightforward way with the common concept of cytotoxicity being due to monomeric PSMs, and the impact of nucleic acids on cytotoxicity being due to lowering of the concentration of that active form by RNA attachment. Further limiting the significance of the findings, whether this interaction has any biological significance on the physiology or infectivity of the PSM producer remains largely unexplored.

We thank the reviewer for the detailed comments. We appreciate the opportunity to further clarify our interpretation of the relationship between PSMα3 assembly, cytotoxicity, and RNA-mediated regulation. In the revised manuscript, and building on the previous revision round, we substantially expanded and refined the Discussion and Introduction to more clearly distinguish between mature fibrils, transient assembly intermediates, and broader assembly state-dependent mechanisms. We also incorporated additional literature representing different perspectives from the field. The revised manuscript presents a model in which biological activity is governed by dynamic assembly pathways and membrane-associated intermediates whose formation, persistence, and structural organization are modulated by environmental conditions, including RNA.

A central point raised by the reviewer is the suggestion that the RNA effects observed here can be explained simply by sequestration of active monomeric PSMα3. We respectfully disagree that this interpretation can account for the data. A monomer-depletion model makes a clear experimental prediction: conditions that promote aggregation should proportionally reduce activity by reducing the free monomer pool. However, our data show the opposite behavior. RNA promotes PSMα3 aggregation, induces liquid–liquid phase separation, and reshapes fibril morphology into distinct polymorphic assemblies, yet preserves cytotoxic and antimicrobial activity over incubation periods during which peptide alone progressively loses activity. Thus, activity does not correlate with suppression of aggregation or maintenance of soluble peptide. Instead, the data indicate that assembly trajectory and supramolecular organization are functionally relevant parameters. We state this point explicitly in the section “RNA preserves PSMα3 bioactivity,” where we added text clarifying that RNA does not prevent aggregation but redirects the assembly pathway toward structurally and functionally distinct states.

To further clarify our interpretation, we substantially revised the section “PSMα3 cytotoxicity arises from dynamic assembly intermediates.” This section now integrates multiple independent lines of evidence supporting an assembly-state-dependent model. Together, these observations argue against a simple binary model in which either monomers alone or mature fibrils alone determine activity. Instead, they support a framework in which transient intermediates formed along the assembly pathway contribute to membrane disruption and cytotoxicity. Consistent with this interpretation, our confocal and super-resolution microscopy experiments directly show PSMα3 accumulation and aggregation at bacterial and cellular membranes (Figs. 5, 6C, S10), supporting a model in which assembly occurs in direct association with membrane interfaces rather than exclusively in bulk solution prior to membrane contact. We expanded the Discussion accordingly.

We acknowledge the reviewer’s alternative interpretation that the nucleolar/nucleic-acid association observed in HeLa cells may reflect post-lysis binding following membrane permeabilization. We agree that this is a valid consideration at the cytotoxic concentrations used here, where membrane disruption is rapid (Figs. 5–6, Movies S1–S2). The Discussion therefore clarifies that nucleolar localization under these conditions is unlikely to represent a distinct intracellular toxic mechanism, but instead reflects the intrinsic nucleic-acid binding capacity of PSMα3 after cellular entry. We accordingly do not claim that intracellular nucleic-acid interactions contribute causally to cell death in these experiments. The potential biological relevance of PSMα3–nucleic acid interactions at sub-cytotoxic concentrations, where membrane disruption does not dominate, remains an important question for future investigation.

We additionally revised the manuscript to clarify the significance of the EGCG comparison. We agree with the reviewer that the EGCG data alone do not demonstrate “amyloid-mediated cytotoxicity,” and we do not make that claim. Rather, the comparison between EGCG and RNA provides evidence that different assembly trajectories produce different functional outcomes. EGCG redirects PSMα3 into amorphous, non-fibrillar assemblies that lose activity, whereas RNA promotes aggregation while preserving activity and generating distinct supramolecular morphologies. If activity depended solely on monomer concentration, both conditions would be expected to reduce activity similarly through sequestration. Instead, the divergent outcomes support the conclusion that assembly architecture and assembly pathway are functionally important.

In response to the reviewer’s concern that the manuscript overstates the concept of “functional amyloid,” we explicitly distinguish between mature fibrils and dynamic assembly processes, and we avoid wording that could be interpreted as implying that mature fibrils themselves are the active cytotoxic entities. At the same time, we note that the broader concept of functional amyloid-like assembly pathways is widely used in biology to describe assemblies whose formation regulates storage, localization, stabilization, or timing of bioactive states, including hormone-storage amyloids, RNA-binding protein assemblies, and bacterial curli systems. Within this framework, our interpretation is that PSMα3 assembly dynamics modulate the availability and lifetime of bioactive species rather than that mature fibrils themselves are directly toxic.

Importantly, we also broadened the manuscript substantially by incorporating independent studies from multiple unrelated systems supporting the principle that supramolecular organization influences biological function. These additions include: studies showing that structured fibrillar assemblies of LL-37 are required for specific antibacterial activities; work demonstrating that the nanoscale organization of β-defensin–nucleic acid complexes governs immunostimulatory potency; studies correlating α-helical solid-state conformations with cytotoxicity across fibril-forming antimicrobial peptides; salt-induced PSMα3 polymorphism studies showing distinct toxicities for amorphous versus fibrillar assemblies; and real-time AFM work demonstrating that membrane-associated protofibrillar intermediates are more disruptive than mature fibrils. We also added discussion of recent cryo-EM structures showing that RNA acts as a structural cofactor shaping tau fibril polymorphism at atomic resolution, as well as two-dimensional infrared spectroscopy studies demonstrating coexistence of cross-α and cross-β PSMα3 polymorphs. Together, these orthogonal observations from multiple systems support the broader principle that assembly architecture is a major determinant of biological behavior.

We also addressed the reviewer’s concern regarding biological relevance. We agree that direct in vivo validation remains an important future direction and state this explicitly in the revised Discussion. However, we respectfully submit that establishing the mechanistic principle that RNA regulates PSMα3 assembly state and functional output is itself a meaningful contribution independent of immediate in vivo confirmation. To better contextualize potential physiological relevance, we expanded the “Biological and therapeutic implications” section to discuss biologically plausible extracellular environments in which PSMα3 may encounter nucleic acids, including biofilms enriched in extracellular RNA, extracellular vesicles, damaged host tissues, inflammatory milieus, and host-derived extracellular RNA released as DAMPs.

Overall, the revised manuscript reflects a substantially expanded discussion of PSMα3 assemblystate-dependent activity, the role of RNA in modulating assembly trajectories, and the broader conceptual implications for membrane-active peptide assemblies.

Further remarks:

(1) Circumstantial evidence based on the "amyloid inhibitor", EGCG: The results with EGCG, which has been shown to have a moderate amyloid-reducing effect on PSMalpha 1 and PSMalpha4, should not be taken as evidence for amyloid-based cytotoxicity. While increased concentrations of EGCG reduced the cytotoxic effect of PSMalpha3, it is not convincingly shown that this is due to a lower concentration of amyloid vs. monomeric PSM.

We agree that the EGCG data alone should not be interpreted as evidence that mature amyloid fibrils are the directly cytotoxic species. Our interpretation is more limited and focuses on the effect of assembly redirection. Specifically, EGCG redirects PSMα3 into amorphous, non-fibrillar assemblies that lose activity, whereas RNA promotes aggregation while preserving activity and producing structurally distinct assemblies. The key conclusion is therefore that functional outcome depends on the nature and trajectory of assembly rather than on aggregation versus non-aggregation alone. We clarified this distinction in the revised Discussion section addressing RNA- versus EGCG-mediated modulation of PSMα3 assembly.

(2) It is appreciated that the authors refrain from presenting the unsubstantiated concept of "functional" PSM amyloids in the discussion. However, wording in that direction must also be removed from other parts of the manuscript (e.g. "bioactive fibrillar polymorphs". "The formation of cross-alpha amyloids has been correlated with toxic activity", etc.), generally refraining from uncritically implying that amyloid formation underlies PSM biological activity, and rather discussing that the much more likely explanation of the findings is a lowering of cytolytically active, monomeric PSM concentration.

In the Introduction, the phrasing 'may enable dynamic switching' has been used to soften the mechanistic claim regarding cross-α assemblies. The phrase 'bioactive fibrillar polymorphs' was revised in the previous round. At the same time, statements such as “cross-α amyloid formation has been correlated with toxic activity” are retained because they describe experimental observations reported in multiple studies (including Tayeb-Fligelman et al., 2017, 2020; Malishev 2018), without implying direct causality (correlation is not causation). We now explicitly frame these observations within a broader discussion of transient assembly intermediates and assembly-state-dependent toxicity.

(3) Discussion: "PSM alpha3 interaction with nucleic acids within human cells ...supports a comparable mechanism...". Delete. Unsubstantiated.

This sentence was removed in the previous revision round and remains absent from the current manuscript.

(4) The authors should cite papers that have argued against their hypothesis and not only their own manuscripts.

We appreciate this suggestion and agree that alternative interpretations should be represented explicitly. In the revised manuscript, we added and discussed studies including Zheng et al. (2018) and Yao et al. (2019) (already cited in both earlier versions), which support models in which advanced amyloid formation reduces cytotoxicity and active species are prefibrillar. These studies are now discussed substantively in both the Introduction and Discussion alongside our own work and that of others.

More broadly, we revised the manuscript to present the current understanding of PSMα3 toxicity as an actively debated question in the field rather than as a settled model. At the same time, we note that citing our prior studies remains necessary where the present work directly builds upon previously reported structural, biophysical, and mechanistic observations.

If the reviewer has additional specific references in mind, we welcome them and will incorporate them.
